# First implantation of a bioprosthetic total artificial heart for a mediastinal paraganglioma

**DOI:** 10.1016/j.jhlto.2026.100485

**Published:** 2026-01-09

**Authors:** Ali Akamkam, Marie-Eve Garcia, Elie Fadel, Julien Guihaire

**Affiliations:** a Department of Cardiovascular Surgery, Marie Lannelongue Hospital, Groupe Hospitalier Paris Saint Joseph, Le Plessis Robinson, France; b Department of Multidisciplinary Oncology and Therapeutic Innovations, Assistance Publique-Hôpitaux de Marseille, Marseille, France; c University of Paris Saclay, Le Kremlin Bicêtre, France; d Department of Thoracic Surgery, Marie Lannelongue Hospital, Groupe Hospitalier Paris Saint Joseph, Le Plessis Robinson, France

**Keywords:** mediastinal tumor, total artificial heart, paraganglioma, heart transplantation

## Abstract

Resection of cardiac-invasive tumors is challenging, as transplantation is not a viable option for patients with cancer. We report the first implantation of the Aeson total artificial heart (TAH) (Carmat, Vélizy-Villacoublay, France), as a bridge to transplant in a patient with a mediastinal paraganglioma. A 54-year-old man presented with restrictive heart failure related to a paraganglioma. The tumor was invading the right ventricle. No tumor reduction was achieved after chemotherapy. A surgical resection of the tumor combined with the implantation of the Aeson TAH was decided. Embolization of the nutrient vessels of the tumor was performed before surgery to decrease hemorrhagic risk. A positron emission tomography scan at 3 months showed no tumor recurrence. The patient was transplanted 6 months after TAH implantation. No severe primary graft dysfunction or acute rejection was observed. However, the patient developed refractory vasoplegia, which ultimately led to multiorgan failure and death 6 months after transplantation.

## Background

Mediastinal paragangliomas are rare neuroendocrine tumors, representing less than 0.3% of mediastinal masses.^1^ Prognosis relies on complete resection (R0), which is difficult or even impossible in the case of cardiac invasion.

The bioprosthetic Aeson (Carmat, Vélizy-Villacoublay, France) total artificial heart (TAH) has recently emerged as a mechanical circulatory support for patients with end-stage biventricular heart failure.^2^ This device is an autoregulated, biocompatible electro-hydraulic pump, equipped with 4 biological valves and 2 outflow conduits. We report the first implantation of a bioprosthetic TAH as a bridge to transplantation for a paraganglioma invading the heart.

## Ethics

Written informed consent and ethical approval were obtained (IRB00012919).

## Clinical findings

**Figure 1:**
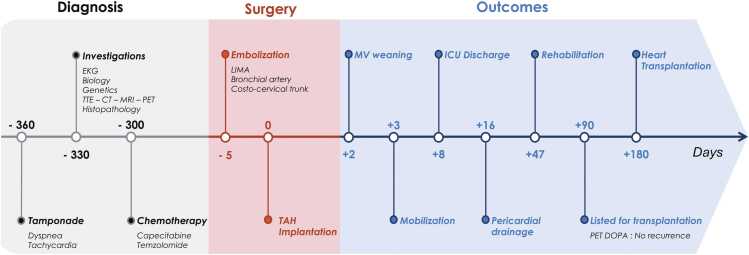
Timeline. CT, chest computed tomography; EKG, electrocardiogram; ICU, intensive care unit; LIMA, left internal mammary artery; MRI, magnetic resonance imaging; MV, mechanical ventilation; PET, positron emission tomography; TAH, total artificial heart; TTE, transthoracic echocardiography.

A 54-year-old man with no personal or family medical history was admitted for a cardiac tamponade related to an anterior mediastinal mass. The patient showed no signs of catecholamine hypersecretion, such as hypertension, sweating, headaches, or palpitations. Electrocardiography revealed an atrial flutter at 132 beats per minute. Transthoracic echocardiography demonstrated reduced left ventricular ejection fraction at 40% (Figure 1).

**Figure 2:**
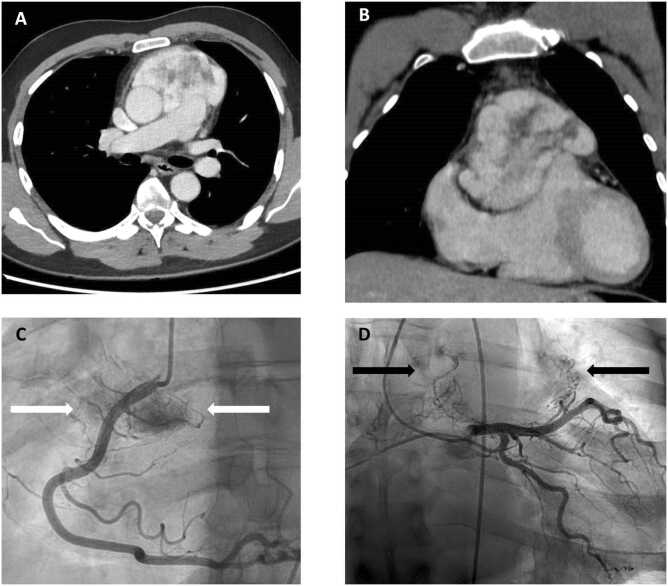
Computed tomography and angiographic assessment of a mediastinal paraganglioma. (A, B) Enhanced chest CT scan imaging showing a highly vascularized tumor of the anterior mediastinum measuring 103 × 65 × 118 mm. The tumor invades the right ventricle, the ascending aorta, and the pulmonary artery. (C, D) Selective coronary angiogram illustrating vascular supply for the tumor. Nutrient vessels originate from the proximal segment of right coronary artery (white arrows, right anterior oblique projection), the left main and left anterior descending arteries (black arrows, right anterior oblique/caudal projection). CT, chest computed tomography.

Chest contrast-enhanced computed tomography (CT) revealed a highly vascularized anterior mediastinal mass (103 × 65 × 118 mm) invading the aorta, the pulmonary trunk, and the right ventricle. The tumor was highly vascularized by coronary and bronchial arteries (Figure 2).

Laboratory testing was notable only for an elevated NT-proBNP at 6,046 pg/ml (reference <125 pg/ml); 24-hour urinary normetanephrine and metanephrine levels were within normal limits.

DOTATOC and 18-FluoroDOPA (18F-DOPA) positron emission tomography/computed tomography (PET/CT) demonstrated intense uptake (standardized uptake value [SUV] max 54.6 and 27.3, respectively), with no evidence of secondary lesions.

A tumor biopsy was performed under CT guidance and confirmed the diagnosis of a paraganglioma. Immunohistochemical analyses showed positivity for chromogranin A and synaptophysin and the absence of cytokeratin and Melan-A expression. The Ki-67 proliferation index was 20%. Constitutional genetic testing did not reveal any germline mutation. The tumor was staged pT3N0M0.^3^

## Treatment

Six cycles of neoadjuvant chemotherapy (28-day intervals) were administered to reduce the tumor size. The protocol consisted of capecitabine (750 mg/m² twice daily for the first 14 days) combined with temozolomide (150 mg/m² on days 10-14 during the first cycle, escalated to 200 mg/m² in subsequent cycles). Treatment was well tolerated with only mild asthenia. After 6 months, CT showed a stable tumor size, with a stable hypermetabolism on DOTATOC PET/CT (SUV max 56).

Despite chemotherapy, tumor compression worsened cardiac function, leading to restrictive heart failure (left ventricular ejection fraction 30%). Surgical resection was ultimately considered the only curative treatment.

Given the high risk of recurrence under immunosuppression, heart transplantation was not considered. The chosen strategy was radical tumor resection combined with implantation of a bioprosthetic TAH as a bridge to transplant. CT confirmed anatomical suitability for implantation of Aeson prosthesis, and the procedure was scheduled. Five days before surgery, nutrient vessels of the tumor (left internal mammary artery, costocervical trunk, and right bronchial artery) were embolized to reduce hemorrhagic risk during surgery.

The pulmonary trunk and the distal ascending aorta were first preserved following careful electrocautery dissection of peritumoral adhesions. Cardiopulmonary bypass was established between the 2 vena cava and aortic arch. Following aortic cross-clamping, the native ventricles were excised en bloc with the tumor. Two atrial cuffs were sewn to the native atrioventricular annuli with a double-layer running suture. TAH was anchored to the atrial cuffs via an interposed titanium interface. Dacron conduits were anastomosed to the aorta and pulmonary artery with pericardium-reinforced running sutures. The TAH driveline was tunneled in the lower right abdominal quadrant. TAH was passively filled and continuously de-aired for 30 minutes. Surgery lasted 11 hours, including 240 minutes of cardiopulmonary bypass.

## Outcomes

Pathological analysis confirmed an extra-adrenal paraganglioma with tumor-free margins. The patient remained in the intensive care unit for 8 days, and mechanical ventilation was weaned on day 2. Mobilization began on day 3, and he was able to walk with assistance. A pericardial effusion was drained on day 16. He was referred to rehabilitation on day 47 with anticoagulation, antiplatelet therapy, and furosemide. This extended observation period was maintained due to the cautious approach associated with the first use of the bioprosthetic TAH at our institution. TAH functioned in automatic mode, maintaining a pump flow above 5 liters/min.

At 3 months, 18F-DOPA PET/CT showed no tumor recurrence, and the patient was listed for heart transplantation. Six months after TAH implantation, heart transplantation was successfully performed. The graft was preserved using a uniform cooling device (Paragonix, Waltham, MA). Total ischemic time was 207 minutes. Macroscopic evaluation of the explanted TAH confirmed the absence of thrombus in the device. The patient was weaned off dobutamine 5 days after transplantation but remained dependent on norepinephrine for the subsequent 6 months. No evidence of graft dysfunction or infectious or immune complications was identified. He subsequently developed multiple organ failure. After discussions with the family, a decision was made to withdraw life-sustaining therapies, and the patient passed away 6 months after the transplantation. No autopsy was performed to identify the cause of the persistent vasoplegia.

## Discussion

Paragangliomas can lead to heart failure through various mechanisms, including restrictive physiology from tumor compression, arrhythmias due to myocardial invasion, stress-related or dilated cardiomyopathy.^4^, ^5^ In our case, the paraganglioma caused restrictive heart failure and atrial flutter, refractory to antiarrhythmics. Beyond the oncologic prognosis, surgical resection was indicated to alleviate these cardiac complications.

To our knowledge, this report shows cardiectomy followed by a bioprosthetic TAH implantation for a paraganglioma invading the heart.

Heart transplantation is generally contraindicated due to the risk of tumor recurrence under immunosuppression. Autotransplantation and complex cardiac reconstruction can be considered for selected cases but remain technically challenging.^6^, ^7^ Finally, TAH allows cardiectomy and R0 resection, providing a tumor-free interval during which patients can demonstrate disease remission before being listed for transplantation.

Only 3 cases of SynCardia (Syncardia, Tucson, AZ) TAH implantation for cardiac tumors have been reported^8^, ^9^, ^10^ (Table 1). However, this device necessitates high-dose anticoagulation, thereby increasing hemorrhagic complications and stroke. In contrast, the bioprosthetic design of the Aeson TAH requires a lower dose of anticoagulation, allowing the use of prophylactic low-molecular-weight heparin and thus avoiding vitamin K antagonist and their associated hemorrhagic risk.^11^

**Table 1:** Case Reports of Total Artificial Heart Implantation for a Cardiac Tumor

	Bruckner et al^8^	Kremer et al^9^	Smail et al^10^
Patient			
Sex	Woman	Man	Woman
Age	32	55	23
Tumor			
Histology	Angiosarcoma	Spindle sarcoma	Spindle sarcoma
Grade	–	High	High
Site	RV TV IVS	LA IAS	RV IVS
Metastasis	0	0	0
Chemotherapy			
Neoadjuvant	Yes^a^	No	Yes^b^
Adjuvant	No	No	No
Surgery			
Previous surgery	Debulking	R1 resection	Debulking
TAH	Syncardia	Syncardia	Syncardia
Heart transplantation	No	No	Yes
Time waiting list	–	–	1 year
Follow-up			
Outcomes	Liver failure	Cerebral bleeding	Cerebral bleeding
Survival	Weeks^c^	10 days	7 days

Abbreviations: IAS, interatrial septum; IVS, interventricular septum; LA, left atrium; R1, partial resection; RV, right ventricle; TAH, total artificial heart; TV, tricuspid valve.

a First line: paclitaxel; second line: adriamycine ifosfamide; third line: doxorubicine, gemcitabine, docetaxel.

b VIDE: vincristine, ifosfamide, doxorubicin, and etoposide.

c The exact time of death was not reported in this case.

There is no consensus on the optimal waiting period between tumor resection and transplantation. Unlike sarcomas, paragangliomas are less aggressive. In our case, we considered 3 months without recurrence sufficient to proceed with transplantation.

## Conclusion

Advances in circulatory mechanical support may shift the paradigm in managing cardiac-invasive tumors by enabling radical surgical resection.

## Author Contributions

**Ali Akamkam:** Investigation, Writing – original draft, Visualization. **Marie-Eve Garcia:** Investigation, Writing – review & editing. **Elie Fadel:** Investigation, Writing – review & editing. **Julien Guihaire:** Conceptualization, Investigation, Writing – review & editing, Supervision.

## Declaration of Competing Interest

The authors declare that they have no known competing financial interests or personal relationships that could have appeared to influence the work reported in this paper.
